# Pharmacogenetic Approaches in Personalized Medicine for Postoperative Pain Management

**DOI:** 10.3390/biomedicines12040729

**Published:** 2024-03-25

**Authors:** Maria Leonor Ferreira do Couto, Sara Fonseca, Daniel Humberto Pozza

**Affiliations:** 1 Experimental Biology Unit, Department of Biomedicine, Faculty of Medicine of Porto, University of Porto, 4200-319 Porto, Portugal; up201907620@edu.med.up.pt; 2 Anesthesiology Department, São João University Hospital Centre, 4200-135 Porto, Portugal; saracunhafonseca@gmail.com; 3 Institute for Research and Innovation in Health and IBMC (i3S), University of Porto, 4200-135 Porto, Portugal

**Keywords:** pharmacogenetics, personalized medicine, postoperative pain, pain management, drug response, genetic markers, therapeutic efficacy

## Abstract

Despite technical and pharmacological advancements in recent years, including optimized therapies and personalized medicine, postoperative pain management remains challenging and sometimes undertreated. This review aims to summarize and update how genotype-guided therapeutics within personalized medicine can enhance postoperative pain management. Several studies in the area have demonstrated that genotype-guided therapy has the ability to lower opioid consumption and improve postoperative pain. Gene mutations, primarily *OPRM1*, *CYP2D6*, *CYP2C9*, *COMT* and *ABCB1*, have been shown to exert nuanced influences on analgesic response and related pharmacological outcomes. This review underscores the integration of pharmacogenetic-guided personalized medicine into perioperative care, particularly when there is uncertainty regarding opioid prescriptions. This approach leads to superior outcomes in terms of postoperative pain relief and reduced morbidity for numerous patients.

## 1. Introduction

Postoperative pain (POP) is a set of unpleasant sensory and emotional experiences that follows surgically induced tissue injury, being associated with physiological and behavioral responses [1,2]. Despite many technical and pharmacological advances over the last years, including pharmacogenetic within personalized medicine and optimized therapies, management of POP is still inadequate. High incidence of POP, around 65%, has been reported in the literature, with the median score for worst pain intensity varying from 5 to 9 on an 11-point numerical rating scale (0–10 NRS) [3,4,5,6,7,8,9,10]. Moreover, pain-induced complications are common and include pulmonary infections, increasing risks of cardiovascular, renal, and gastrointestinal dysfunction, immunodepression, postsurgical infection and poor wound healing [6,8,9,11]. Fear, anxiety, stress, and insomnia are among the psychological, physiological and behavioral responses that characterize the multidimensionality of the postoperative pain experience. It should also be stressed that POP, once established, is more resistant to analgesic treatment [6,8,12].

Despite the possibilities of POP management with different drugs and techniques, pain that persists after the surgical wound has healed is a major and largely unrecognized clinical problem. In fact, POP is followed by persistent pain in 10–50% of individuals in a wide variety of operations [12,13], and the intensity of the POP seems to be associated with the risk and severity of such pain chronification [12,14]. Therefore, ineffective POP management may have short- and long-term consequences, and lead to increased health care costs due to increases in length of stay, opioid abuse, higher morbidity, or residual disability [8,9,15].

Pharmacogenetics holds the promise of enhancing pain management by preemptively predicting an individual’s reaction to a particular analgesic before treatment beginning [16,17,18,19], achieved through analyzing polymorphisms in certain genes associated with altered drug metabolism. Moreover, it acknowledges that genes impacting drug receptors and other pain pathways can also play a significant role.

Over 90% of current medications, including analgesics, are metabolized by the cytochrome P450 (CYP450) enzymes. Thus far, the Human Genome Project has identified 57 *CYP* genes that influence drug metabolism [16,17]. Polymorphisms in *CYP* genes can alter enzyme function, leading to different phenotypes. Currently, we can categorize phenotypic changes in CYP enzymes in four groups: poor metabolizers, intermediate metabolizers, extensive metabolizers (normal) and ultra-rapid metabolizers [17,18]. The *CYP1*, *CYP2*, and *CYP3* gene families are associated with drug metabolism, affecting the body’s response to pain. One of the most studied genes in this family is *CYP2D6*. This enzyme is responsible for the metabolism of commonly prescribed analgesics, such as codeine, tramadol and dihydrocodeine [17]. CYP3A4 has also been found to be involved in opioid metabolism [16]. Another important gene in analgesic metabolism is *CYP2C9*, as it is one of the metabolic enzymes for methadone and many NSAIDs [16,17].

Genes associated with specific drug receptors can also change drug efficacy. One example of this is gene *OPRM1*, which encodes μ-Opioid Receptor. This gene is associated with variable potency and effectiveness of morphine among patients [16].

In this context, pharmacogenetic advancements within personalized medicine offer promising strategies for optimizing therapies and improving outcomes in pain management [19,20]. Pharmacogenetics aims to refine and optimize the prescription process of medications tailored to each person by healthcare practitioners [16,17,19]. Consequently, this will lead to the development of best practice procedures in healthcare, resulting in better assistance and quality of life for the patients [4,6,8,21].

Therefore, this review summarizes and updates how the genotype-guided therapeutics within personalized medicine can enhance postoperative pain management in patients undergoing surgery, approaching important topics such as pain relief, morbidity, and quality of life.

## 2. Postoperative Pain Management and Pharmacogenetics

High percentages of moderate to severe POP can be found in the literature [3,4,5,6,7,8,9], leading to interferences in vital functions, causing adverse health effects, and affecting capacity of recovering [6,7,8,9,11,15]. The best POP management is ethical and imperative [22,23,24] not only for pain relief, but also to avoid the related morbidity with deleterious consequences in the short and long terms. Pre-emptive pharmacogenomic (PGx) testing emerges as an invaluable tool in guiding drug selection and determining the appropriate dosage, including in patients undergoing elective surgery [16,25,26].

**Figure 1:**
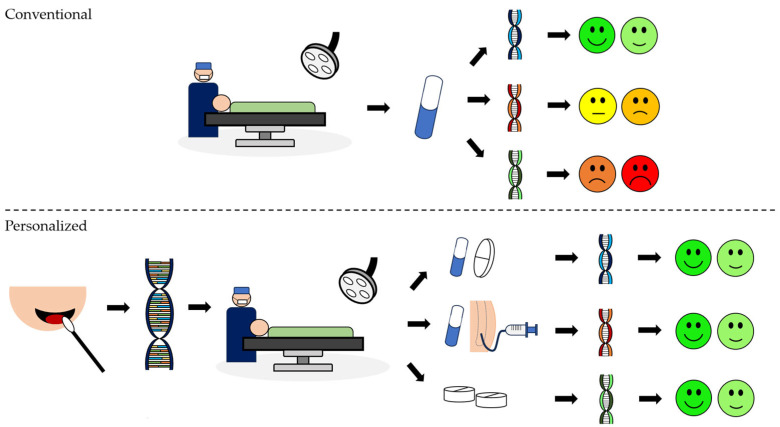
Differences between conventional and personalized analgesic approaches: Conventional approach: The same protocol in a conventional analgesic regimen can elicit different responses in different individuals. Personalized: In a programmed surgery, after an informed consent, buccal swabs provide a convenient means of obtaining a DNA sample. Following the collection, the DNA is extracted. Based on selected genes, the tailored analgesic protocol is developed to suit individual needs and optimize pain management. Legend: two green faces represent none to mild pain; two yellow faces represent mild to moderate pain, and two red faces represent moderate to severe pain.

Genotype-guided therapeutics can lead to a more effective analgesic protocol, enhancing pain management and reducing related complications, particularly in high-risk patients. This procedure involves DNA extraction and evaluation to determine genetic factors relevant to pain response and medication metabolism. The next step is to tailor the analgesic protocol according to the results of the genetic tests, ensuring personalized and optimized pain relief for each patient (Figure 1).

Recent studies have indicated that implementing pharmacogenetic tests can reduce opioid consumption and side effects, together with lowering post-operative pain levels for most patients. Genetic variations, mainly in *OPRM1*, *CYP2D6*, *CYP2C9*, *CYP3A4*, *COMT*, *ABCB1*, and *SLC22A1*, were demonstrated to influence analgesic response, side effects and postoperative chronification, highlighting the importance of personalized medicine in POP management.

## 3. Comprehensive Analysis of Clinical Studies

A comprehensive literature review was conducted and divided into two analyses: 1. controlled clinical trials, and 2. prospective clinical trials. The inclusion criteria applied for the first analysis comprised adult human participants and randomized controlled clinical trials or controlled clinical trials published in English and with at least 10 patients. The inclusion criteria for the second analysis comprised adult human participants and only prospective studies published in English with at least 50 patients. The exclusion criteria for both analyses comprised studies in which participants did not undergo surgical procedures and those that investigated non-postoperative pain.

Three electronic bibliographic databases, Web of Science, PubMed, and Scopus, were used for the manuscript search, and this was carried out between December 2023 and January 2024. The search strategy was built up combining MeSH terms in PubMed and keywords in both Scopus and Web of Science.

The PubMed search yielded 49 articles using the following query: “Pharmacogenetics”[Mesh] AND “Pain, Postoperative”[Mesh]. A total of 47 articles were identified in Scopus using “Pharmacogenetics”, “Postoperative pain” and “Pain Management” as keywords. Also, 61 articles were identified in Web of Science using “Pharmacogenetics” and “Postoperative pain”. An additional manual search found 5 manuscripts.

One of the authors screened titles and abstracts to assess their relevance and alignment with the objective of this study. After this initial selection, a full-text review was conducted, and information from each selected study was extracted, including the characteristics of the participants and the conclusions drawn. Then, these were systematically compared and evaluated, ensuring that only studies with the appropriate methodology and outcomes were included in the systematic review and that the results were valid and reliable.

The data synthesis process was performed by the extraction of the relevant data from each study, including studied population, screened genes, pharmacogenetic approach, intervention, and main results. The other two authors critically reviewed the study selection process. Whenever discrepancies were present, the solution was found through consensus. Kappa test for agreement was 0.90.

**Figure 2:**
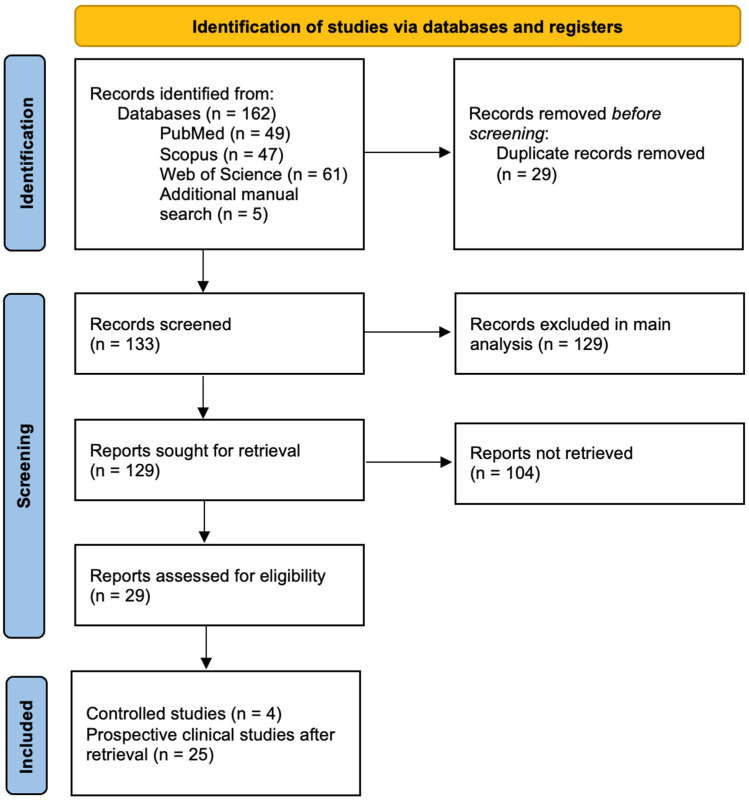
PRISMA flowchart.

The initial search comprised 162 articles, of which 29 were removed since they were duplicated records, making a total of 133 articles. Records were first screened for inclusion in the main analysis, after which reports were retrieved for a secondary analysis of prospective clinical studies. Studies were excluded based on various criteria: study design (*n* = 108), population not meeting inclusion criteria (*n* = 13), assessment of non-post-operative pain (*n* = 4), full text not being available (*n* = 3), and publication language other than English (*n* = 1). For the secondary analysis, 25 non-controlled studies were retrieved, leaving 104 reports unretrieved. Referenced articles that were found pertinent through manual search, despite not being found with the set parameters of the initial search, were also included (Figure 2).

According to the established criteria, three randomized controlled clinical trials and one controlled clinical trial were included for the first analysis, and their descriptions are provided in Table 1.

The main results in Table 1 demonstrated a significantly lower opioid consumption associated with employing genotype-guided therapeutics across all selected studies [27,28,29,30]. This intervention was also associated with lower pain levels in post-operative pain in two studies [27,29]. In the remaining two studies [28,30], no improvement in pain levels was reported. However, Senagore et al. [30] reported a lower incidence of analgesic-related side effects.

Given the limited quantity of controlled clinical studies, subsequent research was conducted, including exclusively prospective clinical trials. The goal was to compile clinical studies involving multiple pharmacogenetic tests for a more comprehensive analysis (Table 2). 

Out of the 29 selected studies, buccal cheek swabs were utilized to collect DNA samples in five studies [27,28,29,30,42], while peripheral blood samples were employed in the remaining 24 studies. DNA extraction technique was not specified in four studies [27,28,29,42].

In the other studies, various DNA extraction methods were utilized: four studies used a conventional phenol–chloroform method [36,46,49,54], four studies employed a Gentra Puregene Blood Kit [34,35,50,51], three studies utilized a QIAamp DNA Blood Mini Kit [43,44,49], two studies used the salting-out method [37,48], two used E.Z.N.A SQ Blood DNA Kit [38,39], while one study each employed a Lab-Aid 820 Midi [31], a MagNA Pure LC DNA Isolation Kit [32], a Wizard Genomic-DNA Purification kit [45], a MagNA Pure LC 2.0 instrument [33], a QIAGEN EZ-1 BioRobot and Blood kit [40], a QIAamp DNA Blood Midi Kit [41], the Guanidinium isothiocyanate method [47], the Chelex method [52], and a PureGene DNA Purification Kit [53].

## 4. Main Genes Related to Postoperative Pain

### 4.1. OPRM1

*OPRM1* encodes the mu opioid receptor, a pivotal drug target. Consequently, the significance of *OPRM1* lies in its important involvement in opioid response and pharmacodynamics, as evidenced by several clinical studies [27,29,30,31,32,40,49,50,51,52,53], with significant improvement in POP outcomes, allowing for reduced opioid dosage and consequently fewer side effects [27,29,30].

It was demonstrated that *OPRM1* mutant homozygote (A118G) patients required more morphine to reach analgesia [50,51,52,53] and reported higher pain scores [50,51]. On the other hand, these mutant homozygote patients were linked to a lower incidence of nausea [50,51] compared to *OPRM1* wild-types (A118A). In one study, both mutant heterozygotes and homozygotes were found to have a better response to tramadol [32]. Mutation carriers were also associated with a higher incidence of opioid side effects [40]. Subjects carrying a IVS3 + A8449G SNP were also found to have a different analgesic response, requiring less fentanyl for post-operative pain control [49].

These findings underscore the potential of *OPRM1* genetic testing to tailor opioid prescriptions for surgical patients, ensuring more effective pain management strategies while minimizing adverse effects based on individual genetic profiles.

### 4.2. CYP2D6

The *CYP2D6* gene encodes an enzyme responsible for metabolizing a wide range of drugs, including antidepressants, antipsychotics, and opioids, affecting their efficacy and toxicity in individuals. Variations in the *CYP2D6* gene can lead to differences in drug metabolism rates, influencing individual responses to medications.

When tailoring analgesic prescriptions based on genetic tests, it is also important to understand if people are poor or rapid metabolizers. Regarding codeine and tramadol, this enzyme has a crucial role, as it is responsible for the conversion to their active metabolites, morphine and O-desmethyltramadol, respectively. In these cases, poor metabolizers may be undermedicated under regular analgesic protocols and may be under increased risk for developing chronic pain due to the less effective POP management [17]. Furthermore, studies focusing on mutant homozygous *CYP2D6* poor metabolizer phenotype patients revealed elevated pain levels [36,43], higher tramadol consumption [34,36,54,55], and an increased need for rescue medication [55]. Notably, mutant homozygous *CYP2D6* patients required higher fentanyl dosages to achieve adequate analgesia [38]. Conversely, ultra-rapid metabolizers will quickly be under high systemic analgesic concentrations, a matter of concern when using opioids due to the high risk of side effects, including respiratory depression and potential for toxicity. It is also important to consider that patients will experience a very strong and short-lasting analgesic effect, and pain returns very fast [17,48]. Additionally, when a breastfeeding mother metabolizes codeine rapidly, it can significantly increase the risk of opioid overdose in infants who are nursing [56,57].

This emphasizes the role of *CYP2D6* genetic testing when planning the use of opioids such as tramadol and fentanyl prescriptions for POP, allowing for more precise pain management strategies based on individual metabolic profiles.

### 4.3. CYP2C, CYP2C19, CYP2C9 and CYP2D6

Despite opioids being frequently measured in genetic tests, other analgesic drugs such as NSAIDs play a crucial role in managing postoperative pain. NSAIDs undergo primary metabolism associated with the CYP2C9 isoenzyme, with metabolizer categories similar to those of CYP2D6. This metabolism pattern can lead to increased plasma concentrations and extended half-life in poor or intermediate metabolizers, elevating the risk of side effects and toxicity.

The *CYP2C* gene locus also plays a complex role in predisposing individuals to peptic ulcer disease (PUD). This predisposition varies depending on the type and dosage of nonsteroidal anti-inflammatory drugs (NSAIDs) used and the patient’s complement of single nucleotide polymorphisms at the *CYP2C* gene locus, including those at *CYP2C9* (where low-activity variants can increase exposure to NSAIDs) and the gain-of-function polymorphism at *CYP2C19* (which enhances the metabolism of gastro-protective arachidonic acid). While CYP2C19 has a minor role in NSAID metabolism, it has been associated with peptic ulcer disease, particularly with the CYP2C19*17 variant, adding complexity and heightening the risk of gastrointestinal side effects, especially in PM patients exposed to NSAIDs for postoperative pain management [58,59,60,61,62,63].

Although not usually used in postoperative pain management, when employing multimodal analgesia, tricyclic antidepressants (TCAs) can be a valuable addition to the treatment arsenal. Amitriptyline, a TCA, undergoes primary metabolism through the CYP2C19 and CYP2D6 pathways. CYP2C19 produces active metabolites such as nortriptyline, while CYP2D6 forms a less active 10-hydroxy metabolite. Variations in these enzymes, such as “CYP2D6 ultrarapid metabolizers” or “CYP2C19 poor metabolizers,” significantly impact drug metabolism. Notably, the metabolism of TCAs, including amitriptyline, involves both CYP2D6 and CYP2C19, highlighting the crucial role of pharmacogenetics in tailoring antidepressant therapy to individual patients [64].

While genetic testing often prioritizes opioids in pain management, the inclusion of other analgesics is crucial for personalized pain management. Thus, genetic testing should encompass a broader spectrum of analgesics, recognizing the advantages of multimodal approaches over single-drug protocols.

### 4.4. ABCB1

The *ABCB1* gene, also known as the multidrug resistance protein 1 (*MDR1*) gene, encodes a membrane transporter protein involved in the efflux of various drugs and toxins from cells, impacting their pharmacokinetics and efficacy. A significant association was demonstrated between the rs9282564 polymorphisms in the *ABCB1* gene and opioid-induced respiratory depression in children. Additionally, *ABCB1* SNP rs2229109 indicated a connection with postoperative morphine doses, suggesting that genetic influences play a role in individual responses to opioids. These associations emphasize the importance of considering multiple genetic factors in tailoring postoperative pain management strategies, especially in pediatric populations undergoing common surgical procedures like tonsillectomy [65].

Increasing evidence suggests that distinct genes and mechanisms play a role in each aspect of opioid response [66]; thus, analgesic effect is a result of the cumulative impact of multiple genes [18].

The impact of *ABCB1* mutations on pain relief with tramadol presents conflicting outcomes. In one investigation [32], patients carrying *ABCB1* mutated alleles demonstrated higher pain relief with tramadol, suggesting a potential correlation between specific genetic variations and enhanced analgesic response. However, in a different study [43], no discernible differences were observed in terms of drug consumption, adverse reactions, rescue analgesic usage, or pain levels among different *ABCB1* genotype subgroups treated with tramadol. This discrepancy highlights the complexity of the genetic factors and others influencing the response to tramadol. It also underscores the need for further elucidation of the underlying mechanisms and variations in patient outcomes.

### 4.5. CYP3

Less directly associated genes may also contribute to enhancing POP management. It was demonstrated that CYP3A4*1G homozygote individuals exhibited a notable pattern, consuming less fentanyl, displaying higher plasma concentrations, and requiring less rescue medication compared to other groups, such as CYP3A4*18, where no significant differences were observed. Furthermore, the interaction between *CYP3A53* and CYP3A4*1G demonstrated an additional layer of complexity, contributing to lower fentanyl consumption [44,46,47]. These insights underscore the potential of CYP3A4*1G and CYP3A4*18 in tailoring pain management strategies.

However, additional studies should explore the broader applicability of the cytochrome P450 gene variations in diverse populations and surgical settings, providing a comprehensive understanding of whether these genes should be included in the panel for effective and reliable personalized pain management strategies. Clarifying the role of these genes through expanded research will contribute crucial insights to guide the development of more targeted and efficient approaches in postoperative pain care.

### 4.6. COMT

The *COMT* gene is located at the gene map locus of 22q11.2 and encodes the enzyme Catechol-O-methyltransferase, responsible for the metabolism of catecholamines, such as adrenaline, noradrenaline and dopamine.

Evidence has demonstrated the significance of numerous *COMT* single nucleotide polymorphisms (SNPs) in enzymatic activity and in modulating an individual’s sensitivity to/perception of pain. Variations in COMT activity have been associated with pain sensitivity haplotypes. The most extensively studied SNP in the *COMT* gene is rs4680, identified as a functional polymorphism. This involves a guanosine (G) to adenosine (A) transition, resulting in a valine (Val) to methionine (Met) amino acid substitution. This substitution may lead to three possible SNP genotypes: GG (Val/Val) genotype, characterized by high enzymatic activity; AA (Met/Met) genotype, associated with defective enzymes; and GA (Val/Met) genotype, demonstrating moderate enzymatic activity [67,68]. In the existing literature, three primary haplotypes have shown a strong correlation with sensitivity to experimental pain [69]. These include low (LPS), average (APS), and high (HPS) pain sensitivity haplotypes.

The combination of *COMT* SNPs rs6269, rs4633, rs4818, and rs4680 determine LPS (GCGG), APS (ATCA) and HPS (ACCG) *COMT* haplotypes. These haplotypes contribute to the individual variation in postoperative opioid consumption: theoretically, under the same noxious stimulus, the greater the sensitivity to pain, the more opioid for analgesia [65].

Interestingly, there are contradictory findings for rs4680. The effects of the *COMT* gene haplotypes on the analgesic doses in pain are not so linear: it was demonstrated that a mutant homozygous rs4680 genotype was associated with higher pain scores [42] and had a lower opioid consumption [35,45]. The mutant G allele of rs4818 was also associated with higher pain levels [35], and mutant homozygous (G/G) were found to have a lower opioid consumption in the post-operative period [42].

Regarding *COMT* haplotypes, contradictory results were found: in one study, it was found that having at least one copy of the LPS haplotype resulted in higher pain scores and opioid consumption [42], whereas another study stated that LPS carriers had lower pain scores [35]. The APS/APS diplotype was also associated with a lower opioid consumption [45]. The HPS haplotype also had conflicting results, having been shown both to increase [39] and to lower opioid consumption [33].

Indeed, the reasons for the varied effects of *COMT* gene haplotypes on analgesic doses during acute and chronic pain remain unclear. It is plausible that conditions such as cancer and other chronic pain types entail prolonged or repetitive pain stimuli, potentially leading to increased neuronal enkephalin consumption and compensatory upregulation of mu opioid receptors. This process may result in the modulation of pain sensitivity, consequently reducing the requirement for opioids [33,39].

The impact of *COMT* haplotypes on pain also appears to vary with gender and ethnic background. Women carrying the *COMT* HPS haplotype (associated with low COMT activity) tend to experience heightened pain and exhibit decreased hepatic COMT levels [31,70], potentially linked to estrogenic levels.

The relationship between gene–gene networks, such as *COMT* and *OPRM1*, may also influence pain susceptibility and the efficacy of opioid analgesics. Patients having AA (Met/Met) of *COMT* rs4680 and AG of *OPRM1* rs1799971 consumed the largest amount of opioid compared to those having other combinations [71]. Additionally, no association between different *COMT* haplotypes and symptoms such as nausea, vomiting, or dizziness was found [39].

Hence, this biomarker could prove invaluable in a multifactorial model integrating biological, physical, and social factors to predict both pain experience and opioid response, all while minimizing potential side effects. Its evaluation should be conducted in conjunction with other genetic and non-genetic factors.

## 5. Precision Personalized Medicine

To mitigate opioid misuse, alongside pharmacogenomic testing, precision personalized medicine for pain management should adopt an individualized approach, optimizing the control of postoperative pain while reducing associated side effects, especially with opioids. This rationale may justify initially targeting high-risk patients for poor pain control or chronification (e.g., major surgeries) for testing, with subsequent expansion to other patient groups [19,26].

Additionally, pain management should include a multimodal approach, since it is safer and better than single drugs in reducing POP, mainly in more invasive surgeries [72,73,74,75]. Evidence-driven multidisciplinary teams ensure optimal POP management with minimal morbidity, promoting enhanced analgesia. Tailored POP protocols aligned with the surgical procedure type effectively reduce pain and analgesic consumption, minimize side effects, discourage the use of non-recommended on-demand analgesics, and expedite recovery [27,28,29,30,72,73,74,76] (Table 2). To achieve this, it is recommended to utilize various analgesic agents, transition between opioids, adjust administration routes, implement patient-controlled analgesia, and administer local anesthetics whenever necessary. Frequent assessment and recording of pain should also be conducted [74,77,78,79,80,81,82].

## 6. Postoperative Pain and Pharmacogenetics Cost-Effectiveness

In addition to reducing POP, multimodal analgesia helps in decreasing the analgesic rate and severity of side effects. Moreover, pharmacy represents a very reduced percentage of the surgery costs, less than 5%. Much higher expenses are involved in the prolonged length of stay in the hospitals due to the undertreated POP that interferes with patients’ function and consequently recovery [9,15], or even worse, leads to chronic postoperative pain [1]. Since POP is more reported in young individuals [4,7,76,83], enhanced pain management can lead to indirect savings by reducing occurrences of work absenteeism. Furthermore, PGx testing will help clinicians choose treatments and doses objectively, rather than relying on only clinical judgement [19,84]. In this context, the expenses associated with genetic tests are relatively minimal when weighed against the substantial toll of adverse effects, hospitalizations, and enduring chronic pain. Moris et al. even suggested that the majority of PGx-guided treatment is cost-effective or cost-saving [85], although studies for analgesic PGx-guided treatment cost effectiveness are still lacking.

Therefore, it is expected that changes in the current model of analgesia, mainly by personalized medicine based on genetic tests, will contribute to better pain management and healthcare cost reductions [19,26]. Genetic material can be collected using saliva, reducing any unnecessary inconvenience for the patient while equipping the clinician with a potent resource to enhance the patient’s care by creating a pain-related gene panel [19]. Understanding these genetic differences becomes paramount, since small variations can represent a huge impact in several individuals’ responses to medications. Adverse drug reactions (ADRs) are noxious, unintended, and mostly due to genetic variations, leading to a huge burden on the healthcare system. ADRs are responsible for a high number of hospitalizations and deaths, as well as huge healthcare costs that cannot be precisely estimated due to poor reporting [78,80,81,86].

This is especially important when there is a family history of adverse reactions or when individuals do not respond favorably to certain medications. Specific populations may harbor higher occurrences of certain PGx variants, like the *CYP2C19* loss-of-function variants that are more frequent in individuals with East Asian, South Asian, or Pacific Islander heritage [87,88]. Furthermore, African Americans report more postoperative pain than Caucasians, indicating widespread disparities. These stem from complex factors like communication, attitudes, and healthcare accessibility [89]. In this situation, PGx testing could benefit patients, allowing for more effective pain management.

Due to the significant impact of individual genetic data on both the individual and their family and descendants, ensuring the ethical use of genetic information becomes of great importance. Ethical concerns regarding PGx testing, such as privacy, confidentiality, discrimination, and incidental findings [90], should be included in PGx guidelines and regulations.

## 7. Platforms and Evidence-Based Protocols

One of the challenges in incorporating pharmacogenetic testing into clinical practice lies in the complexity of translating genetic laboratory test results into practical prescribing decisions for studied medications. To address this, there needs to be a standardized platform where clinicians can obtain evidence-based conclusions. The Clinical Pharmacogenetics Implementation Consortium (CPIC) is one of the currently available tools. CPIC focuses on creating, curating, and posting freely available, peer-reviewed, evidence-based, updated pharmacogenetic guidelines [91].

The CPIC guidelines assign levels to drugs/genes, and based on this score, advise whether genetic testing prior to prescription is recommended. Other resources available are the FDA-approved labels and PharmGKB Clinical Annotation Levels of Evidence, which also give information regarding drug pharmacogenomics. Some of the drug/gene pairs studied in this review, such as Tramadol and *CYP2D6* [92], have been described in CPIC guidelines, which recommend that genetic data should inform drug prescriptions.

Pain management practices are increasingly relying on PGx testing to enhance their approach in determining the most effective relief strategy for patients while reducing risks and avoiding trial-and-error approaches [19]. This shift is especially evident in ongoing research initiatives such as the ImPreSS Trial [93]. Despite the limited evidence, the investigation on the feasibility and utility of pre-emptive PGx testing to guide medication decision-making in real time will contribute with valuable insights to the evolving landscape of personalized pain management strategies.

## 8. Future Directions

While pharmacogenomic tests are valuable, it is crucial not to overlook other significant factors that influence POP management. Patients should be treated comprehensively under personalized medicine to ensure a correct and appropriate analgesic approach. In this context, individuals differently respond to the same medication due to a multitude of factors, including weight and behavioral variations, sociocultural level, physiological function, and drug interaction including metabolism and elimination [17,19,76]. Age and genetic disparities are also important factors in pain management and pain perception [19,25]. POP, which is less frequently reported in the elderly, also correlates with the necessity of lower doses of analgesics such as morphine [4,7,83,94]. This underscores the importance of adjusting analgesic dosages, such as morphine, for elderly patients, acknowledging the impact of age-related factors on drug responses.

Future directions involving advancements in perioperative care through widespread adoption of better pain management strategies and the development of genetically tailored analgesic drugs could enhance healthcare practices. Integrating pharmacogenomics as an additional tool for postoperative pain management is expected to lead to improved outcomes and reduced risks, as demonstrated by several studies.

It is also important to underscore the imperative for conducting pharmacogenomic Genome-Wide Association Studies (GWAS) geared towards pinpointing novel genetic variants intricately associated with analgesic response in a comprehensive and unbiased manner. These initiatives hold significant promise in unlocking invaluable insights into personalized pain management strategies, thereby bolstering patient care and outcomes within the postoperative approach.

Additionally, it is essential to consider the heterogeneity of studies stemming from varying etiologies of postoperative pain, diverse analgesic protocols, and the genes under investigation. Conflicting results in prospective studies further underline the need for more extensive research in this area, emphasizing the importance of continued investigation and collaboration across multidisciplinary teams.

## 9. Conclusions

In summary, there is a need for better POP management. In this context, our study demonstrated the importance of pharmacogenetic-guided personalized medicine protocols for pain management in surgical patients. This approach has the capacity to yield superior outcomes in terms of enhanced pain relief, reduced morbidity, and overall improvement in patient-reported outcomes by improving standard pain management strategies.

Finally, it is expected that the results of this study will foster awareness for the review of educative programs based on the best practice in pain management. Thus, an exceptionally improved quality of life for surgical patients is anticipated, reducing healthcare and socio-economic costs in the near future.

## Figures and Tables

**Table 1:** Concise overview of relevant studies on pharmacogenetics for postoperative pain comparing genotype-guided versus standard care.

Ref.	Participants/Country/Tools	Intervention/Treatment	Pharmacogenetic	Main Results
Hamilton 2022 [27]	- 107 patients scheduled for total joint replacement - Mean age 56.8 ± 11.7 - Country: USA - NRS	- Control group: acetaminophen, oxycodone, tramadol, and celecoxib - Custom group: hydromorphone, meloxicam	16 genes, including *CYP2D6*, *CYP2C9*, *OPRM1*, *CYP3A4* and *CYP1A2*	- Lower pain levels while reducing the consumption of pain medication in genotype-guided group
Thomas 2021 [28]	- 234 patients undergoing total joint arthroplasty - 58.5% female, mean age 67.0 (52–74 years) - Hospital and Orthopedic Clinic - Country: USA - PROMIS	- Control group: standard recommendations - Genotype-guided group: CYP2D6 PM, IM or UM were recommended to avoid tramadol, hydrocodone, codeine and oxycodone and to use an alternative opioid or non-opioid	*CYP2D6*	- Lower opioid consumption in genotype-guided group with similar pain intensity
Hamilton 2020 [29]	- 25 patients scheduled for total knee arthroplasty - 71.0% female, mean age 69.5 (45.7–89.9 years) - Country: USA - NRS	- Control group: ketorolac, acetaminophen, tramadol, hydrocodone, celecoxib - Custom group: custom-medication regimen	7 genes, including *CYP2D6*, *CYP2C9*, *OPRM1*, *CYP3A4* and *CYP1A2*	- Lower opioid consumption and pain levels in genotype-guided group
Senagore 2017 [30]	- 50 patients undergoing laparoscopic colorectal (44) and major ventral hernia surgery (5) - 64% female, mean age 64.5 - Country: USA - OBAS	- Control group: standard drug protocol - Custom group: altered analgesic selection to avoid medications impacted by a genetic variant	9 genes, including *CYP2D6*, *CYP2C9*, *OPRM1*, *CYP3A4* and *CYP1A2*	- 50% reduction in opioid consumption, decreased related side effects in genotype-guided group

Legend: Ref.—reference, %—percentage; USA—United States of America, PROMIS—Patient Reported Outcome Measurement Information System; OBAS—Overall benefit of analgesia score.

**Table 2:** Concise overview of findings from prospective cohort clinical trials on postoperative pain.

Ref.	Participants/Country/Pain Assessm.	Intervention/Treatment	Pharmacogenetic	Main Results
Zhou 2023 [31]	- 101 colorectal cancer patients undergoing laparoscopic radical resection—52.8% (VAS < 4) and 44.44% (VAS ≥ 4) females, mean age 59.53 ± 5.87 (VAS < 4) and 61.67 ± 4.00 (VAS ≥ 4) - University Hospital - Country: China - VAS	2 Groups based on *OPRM1* A118G polymorphisms: wild-type (A/A) and heterozygous (G/A), and mutant homozygous (G/G)	*OPRM1* A118G genotypes	- *OPRM1* A118G wild gene A is a risk factor for VAS ≥ 4 and increased fentanyl dosage
Saiz-Rodríguez 2021 [32]	- 118 patients with moderate to severe pain after dental surgery - 52.5% female, mean age 24.5 ± 4.9 - University Hospital - Country: Spain - VAS	3 Groups: - Ibuprofen - Tramadol - Combination Analysis: metabolizing enzymes, transporters, and receptors	12 genes, including *CYP2C9*, *CYP2C19*, *CYP3A4*, *CYP2D6*, *ABCB1* and *OPRM1*	- CYP2C9 PM had a longer ibuprofen effect, resulting in higher pain relief - CYP2D6 PM had a slower tramadol effect - *ABCB1* mutated alleles had higher pain relief with tramadol - *OPRM1* A118G G allele had better tramadol response
Matic 2020 [33]	- 125 undergoing cardiac surgery - 8.7% female, mean age 64.1 ± 8.3 - Hospital - Country: The Netherlands - NRS	2 Groups based on intraoperative medication: - Fentanyl - Remifentanyl In both groups, results were assessed for a relation with *OPRM1* and *COMT* polymorphisms	*COMT* and *OPRM1*	- Patients with *COMT* high-pain sensitivity haplotype (HPS) in the fentanyl group consumed less postoperative morphine than patients with the average-pain sensitivity haplotype (APS), but not with the low-sensitivity haplotype (LPS) - No associations were found between postoperative opioid consumption and *OPRM1* rs1799971, individual *COMT* SNPs (rs4680, rs4818, and rs4633), or the combined *OPRM1*/*COMT* genotype
Stamer 2016 [34]	- 205 patients scheduled for elective open abdominal or urological surgery - 32.7% female, mean age 57.8 ± 12.8 - University Hospitals - Country: Germany and Switzerland - VAS	3 genotype groups: 0 active *OCT1* allele (OCT1 poor transporter), 1 active *OCT1* allele (heterozygous for 1 inactive allele), and 2 active *OCT1* alleles (OCT1 extensive transporter). 4 CYP2D6 activity groups: CYP2D6 PM, CYP2D6 IM, CYP2D6 EM and CYP2D6 UM	*SLC22A1* (*OCT1*) and *CYP2D6*	- Loss of *OCT1* function resulted in reduced tramadol consumption and increased plasma concentrations of its active metabolite - CYP2D6 PM had significantly higher tramadol consumption in comparison with CP2D6 EM with no active *OCT1* allele
Tan 2016 [35]	- 973 patients undergoing total hysterectomy - 100% female, mean age 47.8 ± 5.3 - Public Hospital - Country: Singapore - VAS	Groups were created based on *COMT* rs4633, rs4818, rs4680 polymorphisms and *COMT* haplotype	*COMT*	- G allele (mutant) of rs4818 was associated with higher pain levels in the 24 h postoperative period - Lower total morphine consumption and weight-adjusted morphine in mutant rs4680 (GA and AA) - LPS haplotype carriers had lower time-averaged VAS scores
Dong 2015 [36]	- 111 patients after elective nephrectomy - 46.8% female, mean age 49.1 ± 13.3 - University Hospital - Country: China - NRS	3 Groups according to CYP2D6*10 allele: wild-type (CYP2D6*1/*1), heterozygous (CYP2D6*1/*10) and mutant homozygous (CYP2D6*10/*10)	*CYP2D6*	- Mutant homozygous (CYP2D6*10/*10) required a higher tramadol consumption, and patients reported higher pain levels at 2 and 4 h postoperatively
Seripa 2015 [37]	- 90 patients undergoing surgical procedures - 57.8% female, mean age 56.7 ± 13.9 - Hospital - Country: Italy - NRS, RSS	4 Groups according to CYP2D6 activity: UM, EM, IM and PM POP treatment was multimodal, including opioids (tramadol, morphine), NSAIDs (ketoprofen) plus metoclopramide, and ranitidine	*CYP2D6*	- Higher mean sedation in IM in early POP (30 min) - Higher mean RSS score in PM, and a minor mean score in UM was also detected, but not significant
Wu 2015 [38]	- 207 patients recovering from radical gastrectomy - 39.6% female, mean age 46 (22–70 years) - University Hospital - Country: China - VAS	3 Groups according to *CYP2D6* polymorphisms: wild-type (W/W), heterozygous (M/W) and mutant homozygous (M/M) Cumulative Analgesic Consumption of Fentanyl during Postoperative Period	*CYP2D6*	- Mutant homozygous showed a relatively poor analgesic effect with a need for higher dosing on the first day after surgery, and a higher VAS score at 6 h postoperatively, compared to wild-types
Zhang 2015 [39]	- 115 patients scheduled for radical gastrectomy - 38.3% female, mean age 52.8 ± 12.3 - University Hospital - Country: China - VAS	Groups were created based on polymorphisms in *COMT* SNPs (rs6269, rs4633, rs4818, rs4680) and their haplotypes	*COMT*	- Higher fentanyl consumption during the first 24 and 48 h in ACCG haplotype (HPS) than other haplotypes
Boswell 2013 [40]	- 158 patients undergoing caesarean section - 100% female, mean age 27.6 ± 5.8 - University Hospital - Country: USA - NRS	2 Groups according to *OPRM1* polymorphisms: wild-type (AA): both heterozygous and mutant homozygous (AG/GG)	*OPRM1*	- Both heterozygous and mutant homozygous patients had a higher incidence of opioid side effects than wild-type homozygous
Candiotti 2013 [41]	- 152 patients undergoing nephrectomy - 38.1% female, mean age 53.71± 16.2 - University Hospital - Country: USA - VAS	3 Groups according to *ABCB1* C3435T genotype: wild-type (CC), heterozygous (CT) and mutant homozygous (TT)	*ABCB1*	- Mutant homozygous had lower opioid consumption than wild-types in the post-operative period - No significant differences in pain scores and side effects were reported
Henker 2013 [42]	- 79 patients undergoing surgery for isolated orthopedic trauma in extremities - 27.8% female, mean age 39 ± 13 - University Hospital - Country: USA - NRS, Presby Sedation scale	Groups were created based on polymorphisms in *OPRM1* A118G rs1799971, rs1799972, *COMT* SNPs (rs6269, rs4633, rs4818, rs4680) and their haplotypes	*COMT* and *OPRM1*	- Lower opioid consumption during the first 45 min post-surgery in mutant homozygous (G/G) rs4818 compared with the other 2 genotypic groups - Mutant homozygous (A/A) rs4680 presented higher pain scores at 15 and 45 min than the other 2 genotypic groups - Carriers of 1 copy of the GCGG haplotype (LPS) were associated with a higher pain score and opioid consumption
Slanar 2012 [43]	- 205 patients scheduled for elective open abdominal or urological surgery - 32.7% female, mean age 57.8 ± 12.8 - University Hospitals - Country: Germany and Switzerland - VAS	3 groups according to *ABCB1* genotype: wild-type (3435CC), heterozygous (3435CT) and mutant homozygous (3435TT) 4 CYP2D6 phenotype groups: CYP2D6 PM, homozygous CYP2D6 EM, heterozygous CYP2D6 EM and CYP2D6 UM	*CYP2D6* and *ABCB1* (*MDR1*)	- Significant pain differences observed in CYP2D6 related to metabolizer type - No differences in drug consumption, adverse reactions, rescue analgesic or pain among the *CYP2D6* or *ABCB1* genotype subgroups
Tan 2012 [44]	- 94 patients scheduled for gynecological laparotomy - 100% female, mean age 39.7 ± 9.9 - University Hospital - Country: Malaysia - VAS	2 Groups according to *CYP3A4* polymorphisms: wild-type (CYP3A4*1/*1) and mutant heterozygous (CYP3A4*1/*18)	*CYP3A4*	- No significant difference was demonstrated between the genotype groups in terms of fentanyl consumption, pain control and adverse effects
De Gregori 2012 [45]	- 109 patients scheduled for major abdominal and urological surgery - 47% female, mean age 61, ranging from 18 to 75 years - University Hospital - Country: Italy - NRS	Groups were created based on polymorphisms in *OPRM1* A118G rs1799971, *COMT* SNPs (rs6269, rs4633, rs4818, rs4680) and their haplotypes, and 7 other *UGT2B7* SNPs	*COMT*, *OPRM1* and *UGT2B7*	- Patients with APS/APS diplotype required the lowest opioid dosage out of all the participants - Mutant homozygous (A/A) rs4680 had a lower opioid consumption than A/G and G/G groups
Zhang 2011 [46]	- 203 patients undergoing gynecological surgery - 100% female, age ranged from 20 to 50 - University Hospital - Country: China - VAS	3 groups according to *CYP3A5* genotype: wild-type (CYP3A5*1/*1), heterozygous (CYP3A5*1/*3) and mutant homozygous (CYP3A5*3/*3)	*CYP3A4* and *CYP3A5*	- CYP3A5*3 polymorphism with no significant difference in analgesic effect and consumption - Interaction between CYP3A5*3 and CYP3A4*1G lead to a lower fentanyl consumption
Yuan 2011 [47]	- 176 patients undergoing lower abdominal surgery - 52.8% female, mean age 45.9 ± 11.1 - University Hospital - Country: China - NRS	3 Groups according to *CYP3A4* polymorphisms: wild-type (*1/*1), heterozygous (*1/*1G) and mutant homozygous (*1G/*1G)	*CYP3A4*	- Plasma fentanyl concentration was significantly lower in wild-type patients (*1/*1) - Patients in group (*1G/*1G) consumed significantly less fentanyl and had a lower percentage needing tramadol rescue medication
Zwisler 2010 [48]	- 270 patients undergoing primarily thyroid surgery or hysterectomy. - NRS	2 Groups according to CYP2D6 activity: 8.9% Poor Metabolizers (PM), 91.1% Extensive Metabolizers (EM).	*CYP2D6* genotypes	- Oxymorphone formation depends on CYP2D6 - No notable differences in oxycodone response or consumption
Fukuda 2009 [49]	- 280 patients undergoing cosmetic orthognathic surgery - 65.3% female, mean age 25.8 ± 7.4 - University Hospital - Country: Japan - VAS	SNP genotypes: A118G and IVS3 + A8449G grouped into major allele homozygote (AA) and combined heterozygote/minor allele homozygote (AG + GG), resulting in 4 genotype groups.	*OPRM1*	- Minor G allele carriers of IVS3 + A8449G SNP needed significantly less fentanyl - Analgesic effects were diminished in individuals with the minor G allele of the A118G SNP
Tan 2009 [50]	- 994 patients undergoing elective caesarian section - 100% female, mean age 32.6 ± 4.8 - Public Hospital - Country: Singapore - NRS	3 Groups according to *OPRM1* polymorphisms: wild-type (AA) heterozygous (AG) and mutant homozygous (GG)	*OPRM1*	- *OPRM1* mutant homozygotes consumed more morphine and reported higher pain scores than 118A carriers, but had a lower nausea score
Sia 2008 [51]	- 588 patients undergoing elective caesarian section - 100% female, mean age 32.5 ± 4.7 - Public Hospital - Country: Singapore - NRS	3 Groups according to *OPRM1* polymorphisms: wild-type A118 homozygous (AA), heterozygous (AG) and mutant G118 homozygous (GG)	*OPRM1*	- Morphine consumption was lowest in the wild-type homozygous - Pain scores were lowest in wild-type homozygous and highest in mutant G118 homozygous - Wild-type homozygous was associated with the highest incidence of nausea
Chou 2006 [52]	- 120 patients undergoing total knee arthroplasty - 81.5% female, mean age 66.4 ± 7.0 - Hospital - Country: Taiwan - VAS	3 Groups according to *OPRM1* polymorphisms: wild-type A118 homozygous (AA), heterozygous (AG) and mutant G118 homozygous (GG)	*OPRM1*	- *OPRM1* mutant homozygotes consumed significantly more morphine
Chou 2006 [53]	- 80 patients undergoing total abdominal hysterectomy - -100% female, mean age 45.7 ± 6.6 - Hospital - Country: Taiwan - NRS	3 Groups according to *OPRM1* polymorphisms: wild-type A118 homozygous (AA), heterozygous (AG) and mutant G118 homozygous (GG)	*OPRM1*	- *OPRM1* mutant homozygous required more morphine doses to achieve adequate pain control in the first 24 h
Wang 2006 [54]	- 63 patients with gastric cancer scheduled for gastrectomy - 52.9% female, mean age 55.2 ± 10.6 - University Hospital - Country: China - NRS	3 Groups were created according to CYP2D6*10 polymorphism: wild-type (Group I), heterozygous (Group II) and mutant homozygous (Group III)	*CYP2D6*	- Higher tramadol consumption in mutant homozygous, while it did not differ in other groups
Stamer 2003 [55]	- 271 patients scheduled for major abdominal surgery - 37.6% female, mean age 52.4 ± 13.2 - University Hospital - Country: Germany - VAS	2 Groups according to CYP2D6 activity: CYP2D6 EM (mutant heterozygous) and CYP2D6 PM (mutant homozygous)	*CYP2D6*	- PM for CYP2D6 showed a lower response rate to POP tramadol analgesia - Higher need for rescue medication in PM phenotype

**Legend**: Ref.—reference, PM—poor metabolizers, IM—intermediate metabolizers, UM—ultra-rapid metabolizers, SNP—single nucleotide polymorphism, NRS—Numeric Pain Rating Scale, VAS—Visual Analog Scale, RSS—Ramsay Sedation Scale, POP—postoperative pain.
